# Individual illness dynamics: An analysis of children with sepsis admitted to the pediatric intensive care unit

**DOI:** 10.1371/journal.pdig.0000019

**Published:** 2022-03-17

**Authors:** Sherry L. Kausch, Brynne Sullivan, Michael C. Spaeder, Jessica Keim-Malpass

**Affiliations:** 1 University of Virginia School of Nursing, Charlottesville, VA, United States of America; 2 Center for Advanced Medical Analytics, University of Virginia, Charlottesville, VA, United States of America; 3 Department of Pediatrics, Division of Neonatology, University of Virginia School of Medicine, Charlottesville, VA, United States of America; 4 Department of Pediatrics, Division of Pediatric Critical Care, University of Virginia School of Medicine, Charlottesville, VA, United States of America; Yonsei University College of Medicine, KOREA, REPUBLIC OF

## Abstract

Illness dynamics and patterns of recovery may be essential features in understanding the critical illness course. We propose a method to characterize individual illness dynamics in patients who experienced sepsis in the pediatric intensive care unit. We defined illness states based on illness severity scores generated from a multi-variable prediction model. For each patient, we calculated transition probabilities to characterize movement among illness states. We calculated the Shannon entropy of the transition probabilities. Using the entropy parameter, we determined phenotypes of illness dynamics based on hierarchical clustering. We also examined the association between individual entropy scores and a composite variable of negative outcomes. Entropy-based clustering identified four illness dynamic phenotypes in a cohort of 164 intensive care unit admissions where at least one sepsis event occurred. Compared to the low-risk phenotype, the high-risk phenotype was defined by the highest entropy values and had the most ill patients as defined by a composite variable of negative outcomes. Entropy was significantly associated with the negative outcome composite variable in a regression analysis. Information-theoretical approaches to characterize illness trajectories offer a novel way of assessing the complexity of a course of illness. Characterizing illness dynamics with entropy offers additional information in conjunction with static assessments of illness severity. Additional attention is needed to test and incorporate novel measures representing the dynamics of illness.

## Author summary

Patterns of illness recovery and decline may be important to understand the illness course during a critical care admission. This paper highlights a novel approach to characterizing these patterns of change in illness severity. We propose that continuous predictive analytic risk scores can be used as a proxy for patient acuity. These scores can be conceptualized as a highly-dimensional time series representing the patient’s illness trajectory during a critical care period. We take an Information-theoretical approach to consider individual illness state transitions. We hypothesize that the entropy associated with illness state transitions is clinically meaningful and represents the complexity of the transitions among states of illness. We found that higher entropy is associated with more negative outcomes during a pediatric critical care admission. This work has implications for expanding methodologies used to characterize heterogeneous illness trajectories in the critical care environment.

## Introduction

More than one-third of children who die in tertiary care pediatric intensive care units (PICUs) have severe sepsis [1]. In addition, survivors of sepsis have increased lengths of hospitalizations and are at risk of long-term complications [2, 3]. For pediatric patients who experience sepsis, a better understanding of illness dynamics throughout the PICU stay may provide insight into illness trajectories, treatment targets, and responsiveness to therapy. The cumulative burden of illness, as well as the time course of ensuing recovery, are critical to understanding short- and long-term outcomes in critically ill pediatric patients [2, 4, 5]. Illness dynamics during the critical care admission itself may be essential to understand the trajectory associated with the illness course. Further, this type of characterization may help foster enhanced communication of prognosis and goals of care [6].

Prediction models applied to continuously monitored physiological data allow for more effective care delivery by predicting clinical events [7]. Real-time analysis of continuous electrocardiogram data, vital signs, laboratory values, and clinical assessment findings in the electronic health record can identify patients at rising risk of sepsis and other clinical events before overt clinical signs [8]. Additionally, continuous predictive analytic risk scores can also be a proxy for patient acuity [9–13]. The risk scores present a succinct derivation of physiologic inputs and may be used to define individual states of illness severity [9, 10, 12]. These physiological scores are assessed serially, updated frequently, and can be conceptualized as a highly-dimensional time series representing the patient’s illness trajectory during a critical care period.

We posit that there is value in understanding the complexity of a child’s illness trajectory during a critical care admission, particularly among children with a diagnosis of sepsis. We consider illness state transitions- derived from illness severity scores generated from a published multi-variable prediction model [8]- as a stochastic process. Therefore, the time series of illness state transitions can be described by a set of transition probabilities. This approach considers illness state transitions as a stochastic process, rather than a deterministic process. Randomness is modeled probabilistically in this system.

Systems-theoretical approaches have been used to examine nonlinear dynamics in physiological systems [14]. Illness dynamics (i.e., characterizing the patterns of recovery and deterioration) contain clinically useful information [15–18]. Moreover, temporal characteristics of illness during the intensive care stay may provide insights beyond those derived from measures of illness severity at a single point in time. We propose characterizing illness dynamics by evaluating the trajectory of illness as a dynamical system. We consider pattern-forming dynamics of a complex system (i.e., illness dynamics) and consider the importance of fluctuations (i.e., illness state changes)- whether stochastic or deterministic in origin- in quantifying the system [19]. This application is not new. In 1983, Beck and Pauker described the construction and utility of Markov chains in medical decision-making [20]. Sonnenberg and Beck further discussed constructing a range of Markov models in medicine [21]. Both highlight the utility of these models in cases where risk changes over time. More recently, Tighe and colleges used Markov chains to describe postoperative pain trajectories in a post-surgical cohort [15]. To characterize dynamics of respiratory symptoms in children, Usemann and colleges characterized pediatric respiratory dynamics using Shannon entropy calculated from Markov chains transition matrices to identify a high-risk respiratory phenotype [16].

We hypothesize that the entropy associated with illness state transitions may be clinically meaningful and represents the complexity of the transitions among various states of physiological illness. We examined: (1) whether we could identify illness dynamic phenotypes based on the entropy of the pattern of illness state transitions, and (2) whether illness courses with higher entropy were associated with more negative outcomes during the PICU admission.

## Methods

The University of Virginia Institutional Review Board approved this retrospective cohort study.

### Study design

Spaeder and colleagues developed a sepsis prediction model for use in the PICU population [8]. This model development study occurred at the University of Virginia Children’s Hospital and included PICU admissions from December 2013 through May 2016 [8]. The model calculated risk scores every 15 minutes for each patient for the duration of their PICU stay. The risk scores represent the fold increase in the risk of developing sepsis in the following 24 hours relative to the average risk of sepsis in the population. The study authors recorded patient age, length of hospitalization, length of time on a ventilator, and mortality (assessed as all in-hospital mortality). Archived data were available for 1,711 admissions involving 1,425 patients.

For each admission where a sepsis event occurred, we obtained the time series of risk scores for that child’s PICU stay. Using sepsis risk scores as markers of illness severity, we constructed transition matrices of the probability of transitioning from any given illness state to another within 30 minutes. We characterized the transition matrices using Shannon entropy [22]. Using the entropy parameter, we determined phenotypes of illness trajectories using hierarchical clustering. We also examined the association between entropy and negative outcomes using a composite variable composed of in-hospital mortality, days of mechanical ventilation, and length of hospital stay. We used R studio version 3.6.2 for analyses. The R package “DescTools” was used to calculate Shannon entropy and the hclust function in the package “stats” was used for the hierarchical cluster analysis. The rms package was used for regression modeling.

### Description of sepsis prediction model

#### Model development

Spaeder et al. developed the sepsis prediction model for use as a continuous risk estimator as well as a sepsis screening alert [8]. The study authors developed a random forest model on all hospital admissions and validated it using cross-validation. Missing data were imputed with median values. Model performance was evaluated using the area under the receiver operating characteristic curve (AUC). Confidence intervals were based on 200 bootstrap runs resampled by admission. The model had an AUC of 0.750 (95% CI: 0.708 to 0.809).

#### Data inputs to the model

**Fig 1:**
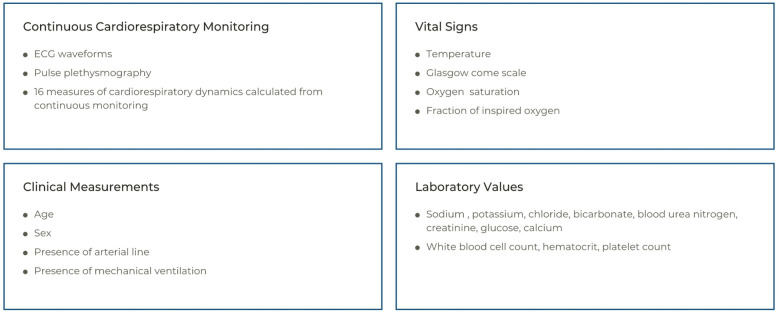
Data inputs to the sepsis prediction model. Inputs to the sepsis prediction model include clinician-entered vital signs, clinical measurements, laboratory values, and features derived from continuous cardiorespiratory monitoring. Features derived from ECG waveforms and pulse plethysmograpgy include the mean and standard deviation of heart rate, respiratory rate (RR), pulse oximetry (SO2), blood pressure, the three pairwise cross-correlations between HR, RR, and SO2, standard deviation of heart inter-beat intervals, local dynamics score (a measure based on the way in which interbeat RR intervals match), local dynamics density of heart inter-beat intervals, coefficient of sample entropy, and the slope of log variance versus log scale between scales 4 and 12 for detrended fluctuation analysis of heart inter-beat intervals.

Inputs to the sepsis prediction model are illustrated in Fig 1. Inputs include (1) continuous cardiorespiratory vital signs (respiratory rate, heart rate, peripheral oxygen saturation, invasive blood pressure, ventilator-measured respiratory rate, and sample-and-hold non-invasive blood pressure) sampled at 0.5 Hz, (2) continuous cardiorespiratory monitoring waveforms (pulse plethysmography and invasive blood pressure tracings sampled at 120 Hz and three leads of ECG sampled at 240 Hz), (3) laboratory measurements (white blood cell count, hematocrit, platelet count, serum sodium, potassium, chloride, bicarbonate, blood urea nitrogen, creatinine, glucose, calcium) and BUN-to-creatinine ratio, (4) clinician-entered vital and clinical signs (temperature, oxygen saturation, Glasgow coma scale, and fraction of inspired oxygen), and (5) clinical covariates (sex, age, presence of an arterial line, and the presence of mechanical ventilation) [8]. Laboratory measurements and clinician-entered vital signs were combined with continuously measured features using sample and hold, and censored when the value was older than 24 hours for vitals and 48 hours for lab values. Moss and colleagues describe the calculation of the cardiorespiratory dynamics measured from the continuous cardiorespiratory monitor [9]. These 16 measures were calculated in 30-minute windows updated every 15 minutes.

#### Sepsis definition

We defined sepsis events using the 2005 International Pediatric Sepsis Consensus Conference criteria [23]. Episodes of sepsis included (1) the presence of systemic inflammatory response syndrome (SIRS) and (2) suspected or proven invasive infection caused by any pathogen. Clinicians individually reviewed charts of every patient who had a blood culture order to establish the time of each sepsis event (i.e., the time of the blood culture order or the time of blood culture collection, whichever came first).

#### Description of data

The model calculated the predicted risk every 15 minutes for each patient, but this study used scores sampled every 30 minutes to account for the fact that the model used the preceding 30 minutes of continuous cardiorespiratory data to generate risk scores. Nonconsecutive risk scores occurred in 767 observations and were removed from the analysis (0.3% of the total data). The remaining scores were adjacent 30-minute score pairs. All risk scores were labeled with the corresponding time in minutes following the start of PICU admission. Actual times were not included; times following admission for each patient were used to obtain scores in the correct time order for this Markov chain implementation.

### Characterization of Markov chains

#### Transition matrix construction

Risk scores generated from the model represent the fold-increase in developing sepsis compared to the average risk of developing sepsis in the study population. For example, a risk score of 2 indicates twice the average risk of sepsis. Risk scores ranged from 0 and 8. We binned scores into four groups to create discrete illness states with clinical meaning. The lowest illness state, 0, has scores in the range [0,1). Illness state 1 has scores in the range [1,2), representing those with an increased risk. Scores in the range [2,3) compose illness state 2 and represent a higher illness state than the preceding states of 0 and 1. The highest risk illness state, 3, contains scores 3 or higher.

Binning continuous scores results in loss of resolution. However, it is necessary to have discrete illness states to model transitions using the implementation of Markov chains that we chose. We examined different binning of illness scores to explore if the results were an artifact of how we defined illness states. We re-defined illness states based on four equiprobable bins (i.e., bin sizes were based on the total number of observed transition states for all admissions. There are an equal number of observations in each bin). We then recalculated the entropy and explored the entropy distribution.

**Fig 2:**
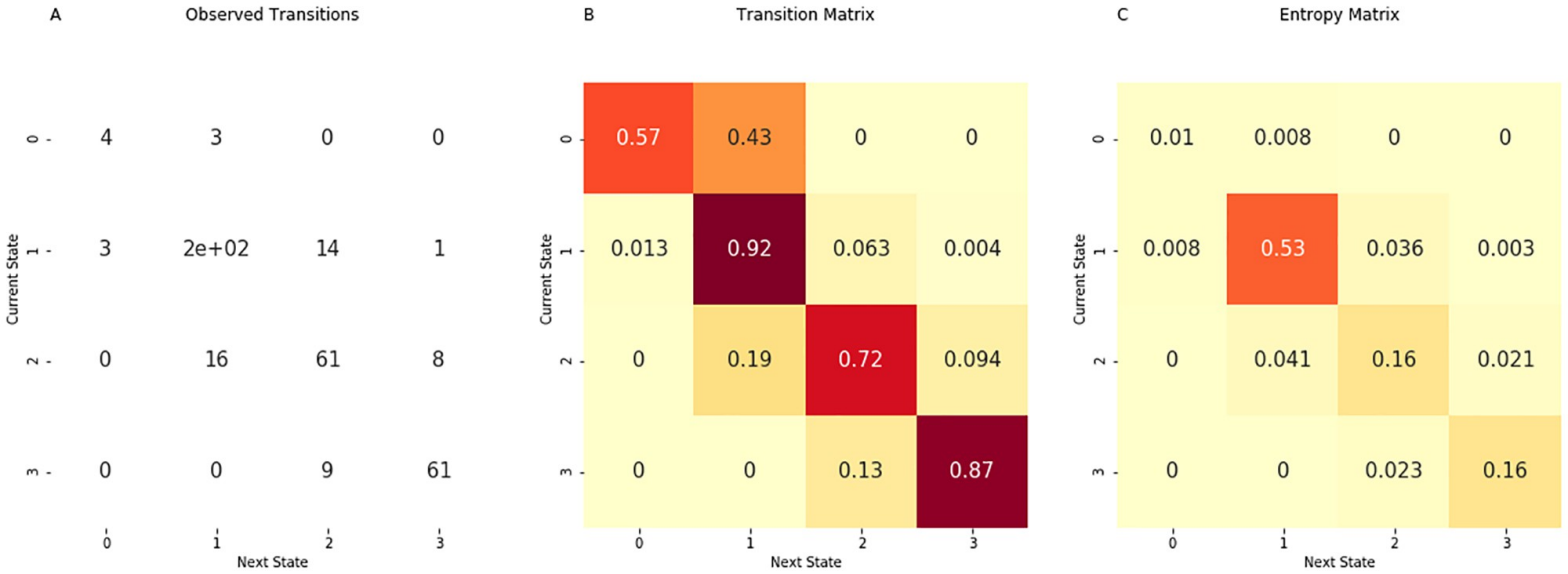
Matrix construction. We counted how often a transition to any illness state occurred for each of the four illness states. The count information is displayed in (A). In the Markov chain transition matrix (B), the probabilities are obtained by dividing the counts by the total number of observations from each initial illness state. In the ‘entropy matrix’ (C), the probabilities are obtained by dividing the counts by the total number of observed transitions. Thus, values in the entropy matrix indicate the density of the observed transitions. The visual difference between the transition and entropy matrices arises from the fact that the row values sum to one in the transition matrix while all the cell values sum to one in the entropy matrix. The figure may be interpreted as follows. The darkest cell in the entropy matrix (c) is in the second row and the second column, where 53% of observed transitions occurred. The corresponding cell in the transition matrix (B) signifies that 92% of the time the patient was in illness state 1, he remained in illness state 1.

For each admission, we used the time series of illness states to create Markov chain transition matrices. The transition matrix is created by row; each row contains the probability of transitioning to a subsequent illness state based on an initial illness state. The number of transitions from each of the four initial illness states to subsequent illness states are counted and inserted into the corresponding cell in the matrix. Each cell in the row is divided by the sum of the transition counts for that row (e.g., Fig 2(A) and 2(B)).

#### ‘Entropy matrix’ construction

Entropy can be considered as a measure of disorder or complexity within a system [24]. The Shannon entropy of a random variable is [22]:

$$\begin{array}{c} H=-\sum p(x)\;log\;p(x) \end{array}$$ (1)

Distributions that are uniform will have higher entropy relative to distributions peaked around only a few values. We conceptualize entropy as giving a quantitative measure of the irregularity in the pattern of illness deterioration and recovery of each child.

To calculate the ‘entropy matrix,’ we recalculate the transition matrix to create a matrix where all the cells sum to one. In this matrix, we divide the number of observed transitions in each matrix cell by the total number of observed transitions (e.g., Fig 1(C)). For each of the 164 PICU admissions, we calculated an entropy matrix. Using the values in each cell of the entropy matrix, we calculated the Shannon entropy of these 164 matrices. We use natural logarithms to define entropy.

### Statistical analysis

We created a composite variable to capture adverse events and increased therapeutic intensity of treatment occurring during a PICU admission. A three-component composite score for each of the 164 PICU admissions was calculated by summing the ranks of the individual components: mortality, days on a ventilator, and days of hospital stay [25]. This composite variable is defined such that larger values are worse than smaller values. Higher rank scores were given to admissions where the patient died, receiving a score of 138. Admissions where the patient survived received a score of one. The ranked values for days on a ventilator and length of stay ranged from one (never ventilated or having the shortest length of stay) to 164. Tied ranks all received the lowest rank value. The composite variable, summed across the three ranked components, ranged from four to 464.

We used the entropy parameter to identify phenotypes of illness dynamics. We clustered the entropy values using an agglomerative hierarchical clustering algorithm. The separation between entropy values was calculated using Euclidean distance. We used Ward’s method, which minimizes the total within-cluster variance, to measure dissimilarity between clusters. We selected clusters based on ease of interpretation and the height of the fusion (on the vertical axis). To evaluate differences across phenotypes, we used Chi-squared tests, Kruskal-Wallis tests when assumptions of normality were not met, and analysis of variance (ANOVA) when assumptions of normality were met.

We examined univariate associations using a nonparametric regression (loess, a moving least squares) to determine the relationship between entropy and the negative outcome composite variable. Using multiple linear regression, we examined the association between entropy with the negative outcome composite variable, adjusting for mean illness score.

## Results

Sepsis occurred during 164 of the 1,711 (9.6%) PICU admissions comprising 144 patients. Demographic information of the cohort is given in Table 1. There were 27 (16.5%) events of in-hospital mortality. The median age was 1.7 years (25% 3.6 months, 75% 7.0 years). The median length of stay in the PICU was 13.9 days (25% 5.0 days, 75% 44.7 days).

**Table 1:** Characteristics of the study population.

Characteristic	n(%)	Mean±SD	Median (IQR)	Range
Male sex	84 (51)			
In-hospital mortality	27(16.5)			
Age (years)		4.3±5.3	1.7(0.3–7.0)	0–17.9
Ventilator days		17.4±30.5	5.1(0.7–20.4)	0–176.8
Hospital stay (days)		46.1±55.1	24.5(8–65)	1–294
ICU stay (days)		30.3±40.1	13.9(5.0–44.7)	1–247
Negative outcomes (composite)		184±116	184(85–268)	4–464

Data are derived from 164 PICU admissions comprising 144 patients. The negative outcomes composite variable incorporates ventilator days, hospital days, and in-hospital mortality. IQR: interquartile range, SD: standard deviation.

### Individual illness state trajectories

**Fig 3:**
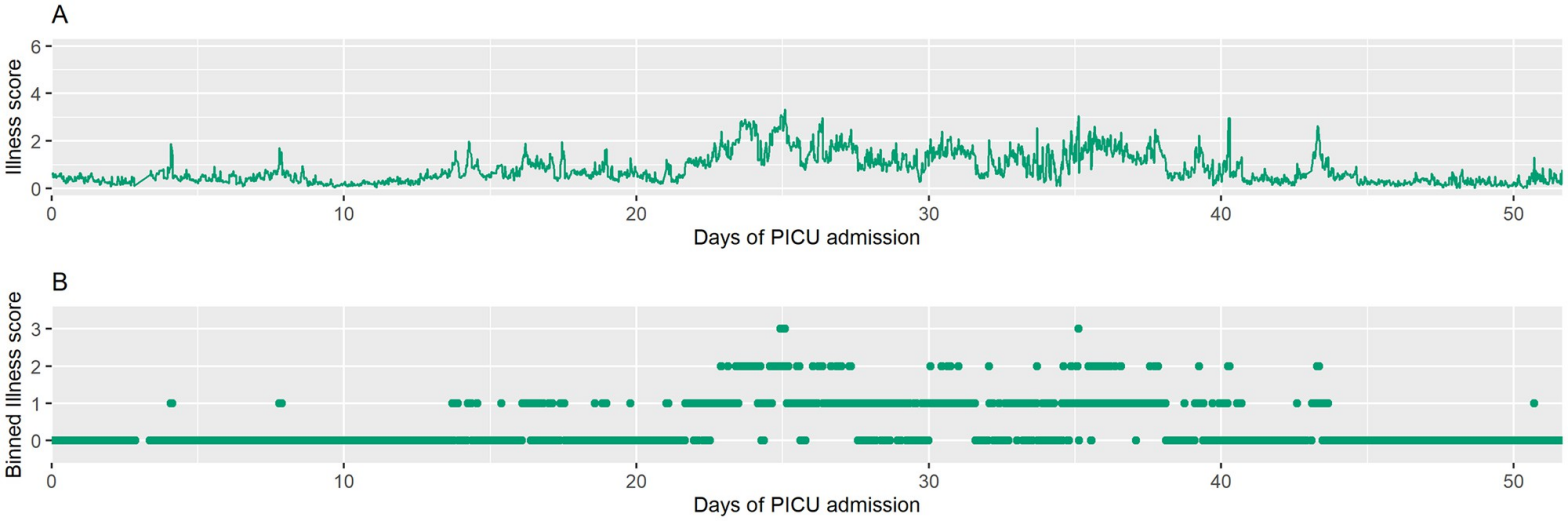
Time series of illness scores for patient 1. (A) Raw time series of illness scores for patient 1. (B) Time series with illness states sampled every 30 minutes and binned to create 4 discrete illness states (0–3). Patient 1 had 2,441 observed illness states, measured every 30 minutes, over a 51-day PICU admission. This patient required mechanical ventilation for 26 days. The average illness score over the duration of her PICU admission was 0.8, and she had a relatively low entropy of 1.44 (range 0 to 2.77).

**Fig 4:**
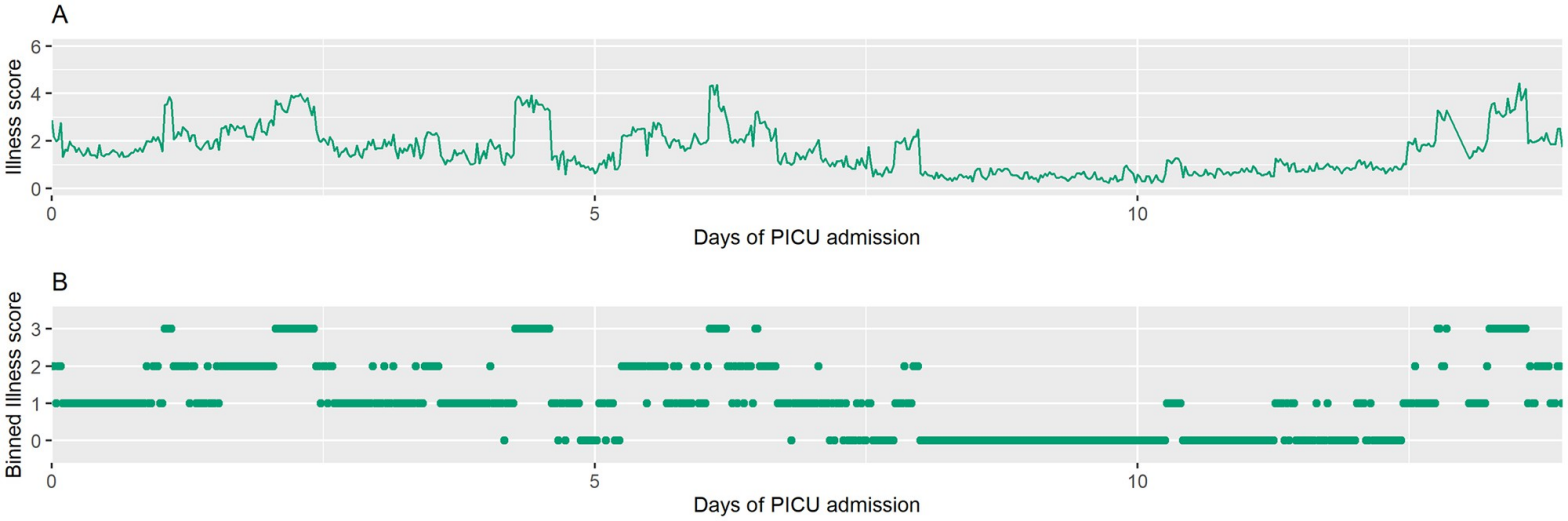
Time series of illness scores for patient 2. (A) Raw time series of illness scores for patient 2. (B) Time series with illness states sampled every 30 minutes and binned to create 4 discrete illness states (0–3). Patient 2 had 659 observed illness states, measured every 30 minutes, over a 14-day PICU admission. Patient 2 required mechanical ventilation for seven days. He had a relatively high entropy of 2.02 and a mean illness score of 1.6.

**Fig 5:**
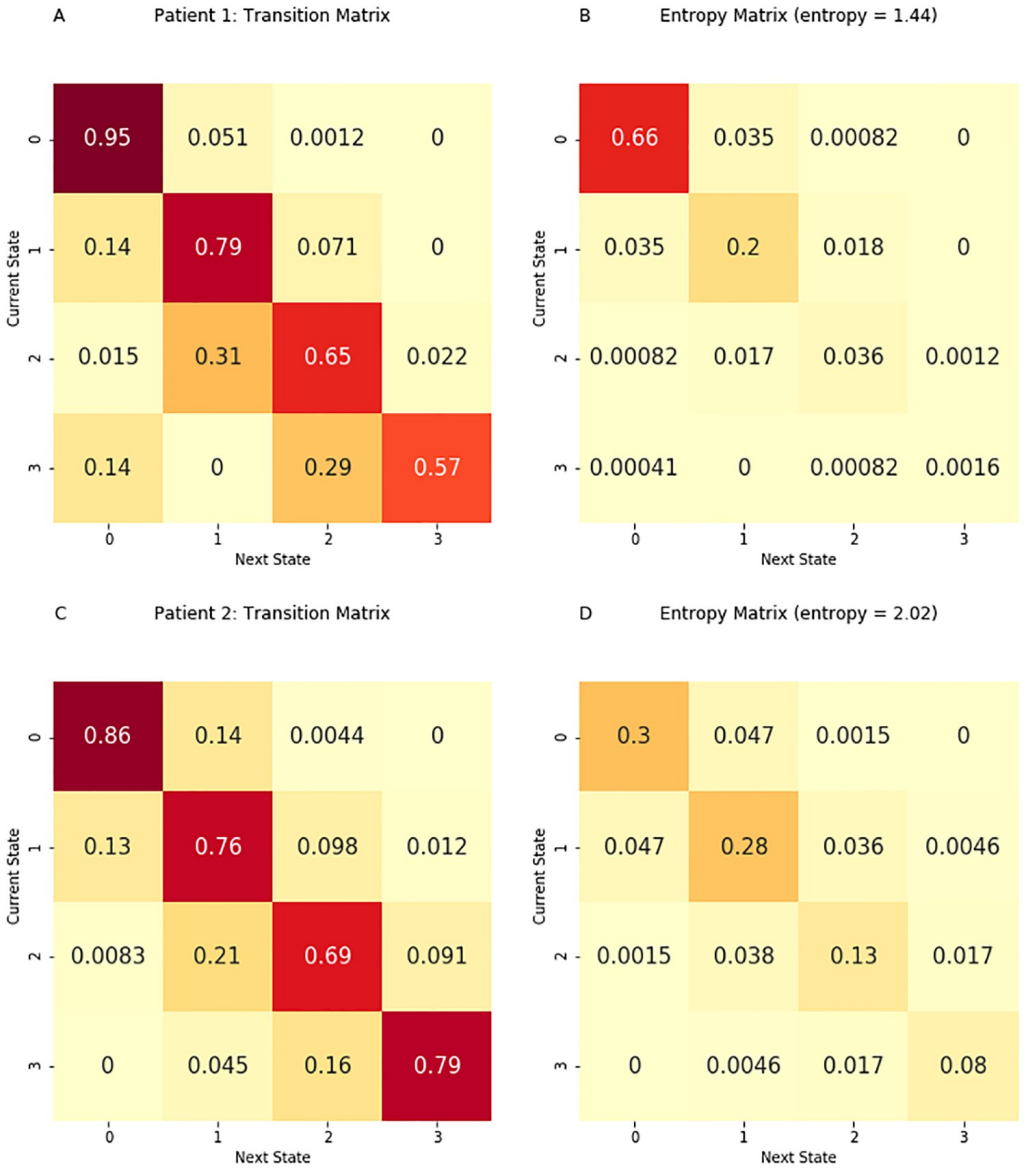
Transition and entropy matrices. The transition matrices for patient 1 (A) and patient 2 (C) show the probabilities of transitioning from a current illness state, denoted by rows, to the subsequent state, denoted by columns. In the entropy matrices for patient 1 (B) and patient 2 (D), the probabilities are normalized from all initial illness states. The figure may be interpreted as follows. The entropy matrix for patient 2 (D) corresponds to an entropy of 2.02, higher than the entropy of 1.44 for patient 1 (B). Patient 2 has a more uniform distribution of transition probabilities than patient 1, whose distribution of probabilities is steeply peaked in row 1, column 1.

We created transition matrices and entropy matrices for each of the 164 admissions where sepsis occurred. Figs 3 and 4 show the raw time series of illness scores that occurred during two representative PICU admissions and the corresponding time series of illness scores after the scores were categorized into 4 discrete illness states. Fig 5 displays the transition matrices and entropy matrices (i.e., the matrix on which the entropy parameter is calculated) corresponding to the two patients’ times series of illness states.

For a point of reference (see Fig 3), Patient 1, a one-day-old female, spent 66 days in the hospital before being discharged. She spent 51 of those days in the PICU, and she required mechanical ventilation for 26 days. Her average illness score over the duration of her PICU admission was 0.8, and she had a relatively low entropy of 1.44 (range 0 to 2.77) for her PICU stay. Patient 2 (see Fig 4), a 3-month-old male, had a 24-day hospitalization before being discharged. He spent 14 days in the PICU and required mechanical ventilation for seven days. He had a relatively high entropy of 2.02 and a mean illness score of 1.6. Even with a shorter PICU stay, patient 2 experienced more fluctuation between illness states, a feature captured by the entropy value (see Fig 5). Each of the remaining PICU admission trajectories was characterized in the same way with a resulting entropy score calculated from the entropty matrix.

### Phenotypes of illness dynamics

**Fig 6:**
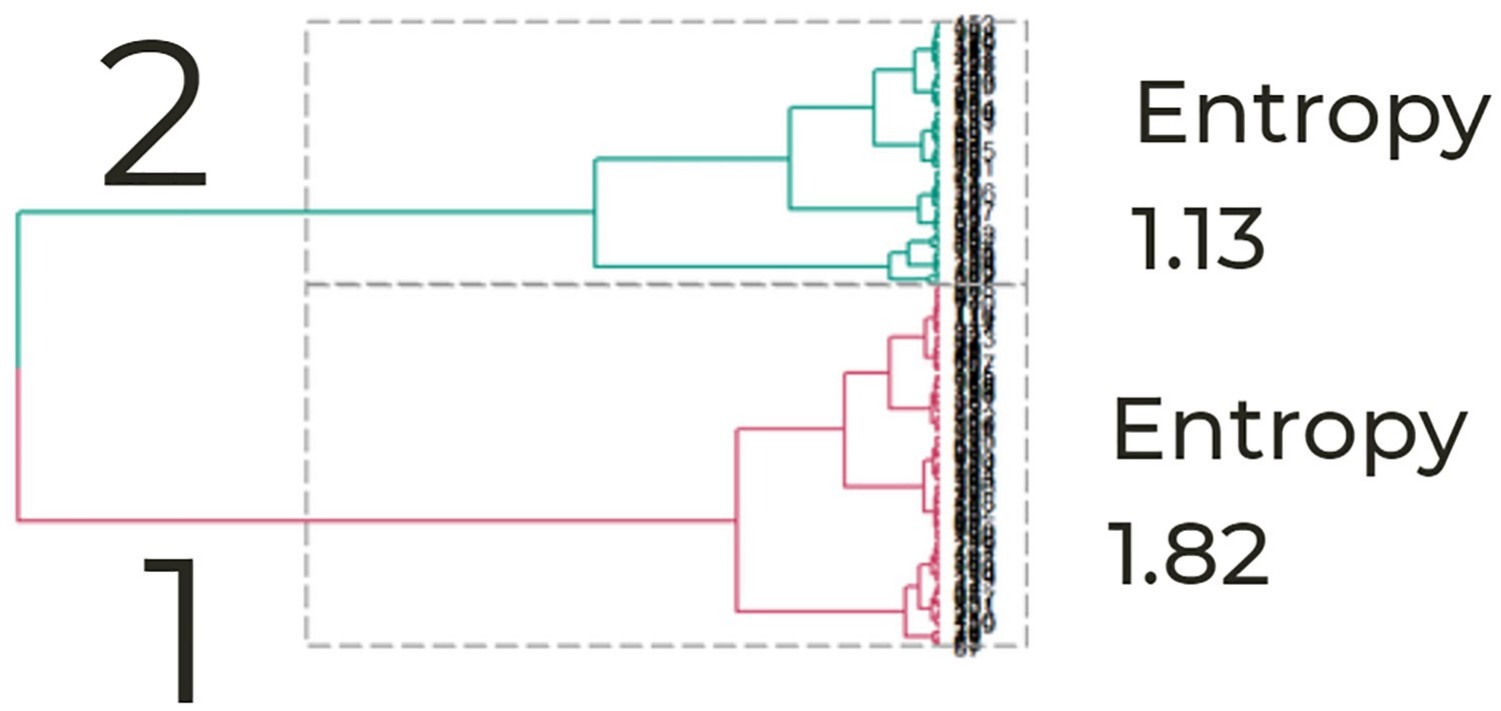
Phenotypes: Two clusters. Hierarchical Ward’s clustering and identified two phenotypes based on the first split. Phenotype one was the “high” entropy cluster. Admissions in phenotype 1 had a higher mean illness score and a higher composite score representing higher illness severity.

To test the hypothesis that entropy-based clustering accurately describes a high- and low-risk group, we used Hierarchical Ward’s clustering and identified two phenotypes based on the first split (see Fig 6). The phenotypes included 95 and 69 admissions (see Table 2). Phenotype one was the “high” entropy cluster (phenotype 1 has an average entropy of 1.82 and phenotype 2 had an average entropy of 1.13). Admissions in phenotype 1 had a higher mean illness score and a higher composite score representing higher illness severity.

**Table 2:** Characteristics of dynamic phenotypes: Two clusters.

	Phenotype 1 (n = 95)	Phenotype 2 (n = 69)	p-value
**Illness severity scores**			
Mean score of entire admission	1.88±0.5	1.81±1.34	**0.002**
**Illness severity transition states**			
Entropy of entire admission	1.82±0.16	1.13±0.36	
**Characteristics**			
Male sex	46	55	0.495
Age (years)	3.97±4.97	4.75±5.80	0.791
Ventilator days	22.3±35	10.6±21.6	
Hospital stay (days)	55±62	33.8±41	
In-hospital mortality	20	11.6	
**Composite variable**			
Negative outcomes	212±113	147±110	**< 0.001** **

Data are presented as % or mean±SD. Phenotypes were defined by Shannon entropy. Differences in the distribution of characteristics across phenotypes were assessed using Chi-squared tests for categorical variables.

* Kruskal–Wallis tests for continuous variables when assumptions of homogeneity of variance and normality were not met. *α*- level of 0.017 are considered significant p -values with the Bonferroni correction.

**One way ANOVA for testing differences between group means when test assumptions were met.

**Fig 7:**
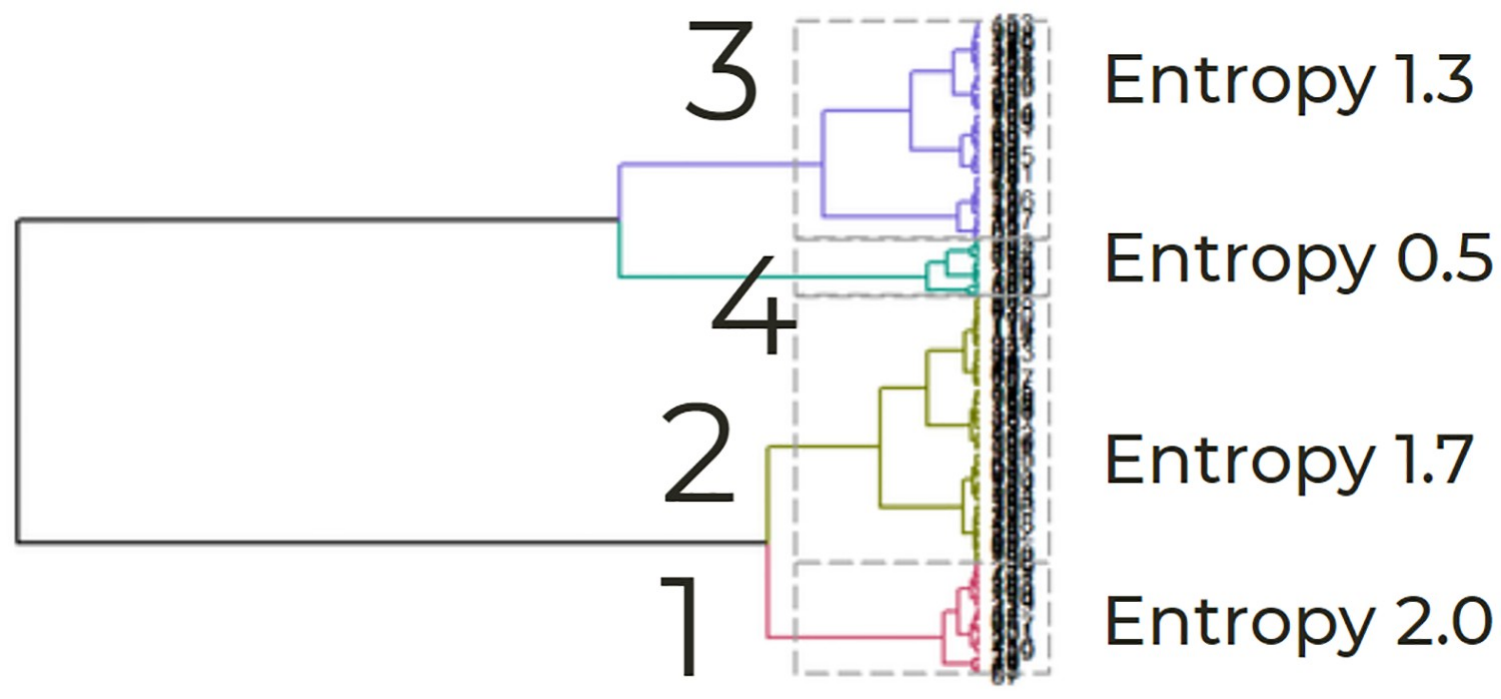
Phenotypes: Four clusters. Allowing for one additional split in Hierarchical Ward’s clustering identified four phenotypes. Phenotype 1, the highest entropy phenotype, characterized the admissions with the worst outcomes (phenotype 1 in this split contains 28 of the original 95 admissions of phenotype 1 based on a two-group cluster).

Given that we had an adequate number of admissions in each of the two phenotypes, we asked if we could further sub-cluster based on entropy in order to identify a phenotype that is significantly worse overall. We examined four illness trajectory phenotypes by allowing for one additional split in Hierarchical Ward’s clustering (see Fig 7). These four phenotypes included 28, 67, 55, and 14 admissions, respectively (see Table 3). Entropy differed across phenotypes (phenotype 1 had the highest entropy of 2.0, phenotype 2 had an entropy of 1.7, phenotype 3 of 1.3, and phenotype 4 had the lowest entropy of 0.5). Phenotype 1, the highest entropy phenotype, characterized the admissions with the worst outcomes (phenotype 1 in this split contains 28 of the original 95 admissions of phenotype 1 based on a two-group cluster).

**Table 3:** Characteristics of dynamic phenotypes: Four clusters.

	Phenotype 1	Phenotype 2	Phenotype 3	Phenotype 4	p-value
	(n = 28)	(n = 67)	(n = 55)	(n = 14)	
**Illness severity scores**					
Mean score of entire admission	1.9±0.3	1.9±0.6	1.7±1.0	2.4±2.3	0.017*
**Illness score transition**					
Entropy of entire admission	2.0±0.1	1.7±0.1	1.3±0.2	0.5±0.2	
**Characteristics**					
Male sex	54	46	58	43	0.536
Age (years)	3.9±4.9	3.9±5.0	4.8±6.1	4.3±4.4	0.992*
Ventilator days	31.7±41.3	18.3±31.4	13.1±23.6	1.1±1.6	
Hospital stay (days)	77.1±77.8	45.8±52.2	39.1±44.1	12.9±10.8	
In-hospital mortality	21.4	19.4	14.5	0	
**Composite variable**					
Negative outcomes	239±119	200±109	168±112	65.7±55	**< 0.001** **

Data are presented as % or mean±SD. Phenotypes were defined by Shannon entropy. Differences in the distribution of characteristics across phenotypes were assessed using Chi-squared tests for categorical variables.

* Kruskal–Wallis tests for continuous variables when assumptions of homogeneity of variance and normality were not met. *α*- level of 0.012 are considered significant p -values with the Bonferroni correction.

**One way ANOVA for testing differences between group means when test assumptions were met.

An ANOVA on the composite variable of negative outcomes yielded significant variation among phenotypes [F(3, 160) = 8.86, *p* < 0.001]. A post hoc Tukey test showed that phenotype 1 had a significantly higher score than phenotype 3 or phenotype 4. There were no significant differences between phenotypes 2 and 3. Phenotype 4 included admissions with no mortality and the fewest ventilator days. Phenotype 1, characterized by the higher mortality, longer hospital stay, and more ventilator days, represents patients considered to be at the highest-risk of negative outcomes.

### Regression for exploration of trends

**Fig 8:**
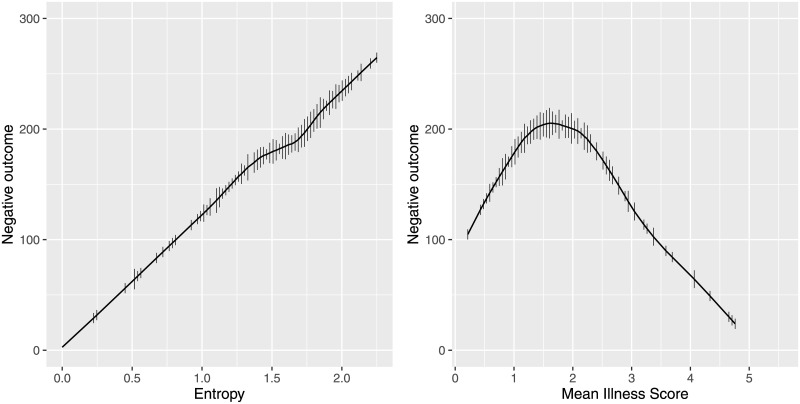
Nonparametric regression. Negative outcome scores as a function of (A) entropy and (B) illness scores. Nonparametric regression (loess, a moving least squares linear regression smoother) estimates of the relationship between the composite variable negative outcomes and the independent variables. Tick marks depict the mean illness scores and entropy distributions. Entropy was significantly associated with the negative outcome composite variable in a univariate analysis (*β* = 107.6, 95% CI 69.0–146.1; *p* < 0.001, R2 = 0.16). After controlling for the mean illness score, entropy remained significantly associated with negative outcomes (*β* = 92.8, 95% CI 23.0–162.6; *p* = 0.009, R2 = 0.20).

We used nonparametric regression to visualize the relationship between entropy and illness scores and the negative outcome composite variable. Entropy does have a linear relationship with negative outcomes, while illness score does not (see Fig 8). S1 Fig displays the relationship between the entropy and the mean illness score.

Entropy was significantly associated with the negative outcome composite variable in a univariate analysis (*β* = 107.6, 95% CI 69.0–146.1; *p* < 0.001, R2 = 0.16). We used restricted cubic splines regression for illness scores, given the nonlinear relationship with the outcome of interest. The association between illness scores and negative outcomes was evaluated with a restricted cubic spline curve with 3 knots (0.85, 1.65, 2.95). After controlling for the mean illness score, entropy remained significantly associated with negative outcomes (*β* = 92.8, 95% CI 23.0–162.6; *p* = 0.009, R2 = 0.20).

#### Sensitivity analysis

We assessed the robustness of our findings by considering different bins to define illness states. There were 201,180 observed illness states for the 164 PICU admission where sepsis occurred. To test whether our findings resulted from our binning of illness states, we examined the entropy resulting from illness state categorization based on equal-sized bins. To create discrete illness states with equal probability of occurring, we again binned scores into four groups. However, in this categorization, the lowest illness state, 0, has scores in the range [0,0.88). Illness state 1 has scores in the range [0.88, 1.45). Scores in the range [1.45, 2.23) compose illness state 2. Illness state 3 contains scores of 2.23 or higher. There are approximately 50,000 illness state observations in each of the four bins. Entropy ranged from 0 to 2.3 using this definition of illness states. The entropy for illness states defined by equal-sized bins was slightly higher (M = 1.6, SD = 0.5) than the entropy for our original illness states (M = 1.5, SD = 0.4).

## Discussion

We present a characterization of individuals’ illness dynamics during the critical care period for children diagnosed with sepsis. We assessed the trajectory of critical illness using illness severity risk scores from a sepsis prediction model to define physiological states. Entropy-based clustering accurately identified a high- and a low-risk phenotype. We were able to sub-cluster one more time and found phenotypes that further stratified the risk of negative outcomes. Based on this stratification, we were able to identify one sub-cluster containing the worst outcomes. This cluster with the highest entropy had the highest risk as estimated by the components of the composite outcome. We also modeled linear regression on the composite outcome that captures cumulative surrogates of illness severity: death, longer ventilator support, and longer recovery times. Entropy was associated with more negative outcomes even after accounting for the mean illness score.

Accurate assessments of illness severity in intensive care settings can help clinicians decide when to initiate or de-escalate therapies, prognosticate the course of illness, and anticipate goals of care. Prognostication in pediatric intensive care settings remains a challenge, and most methods are focused on static periods of time (i.e., first 24 hours of admission or in the hours preceding a sepsis event) [10, 26]. In recent years, there has been increased attention paid to the trajectory of critical illness for children in terms of both short-term outcomes (i.e., sepsis events, mortality) and long-term clinical and functional sequelae [2]. Our findings suggest that entropy may be a valuable parameter to consider and may provide information about illness dynamics that can be assessed in conjunction with static assessments of illness severity. It may be relevant for our understanding of critical illness trajectories in the PICU, particularly among children with long lengths of stay. The developed method may quantify illness severity patterns and could be considered for future use in evaluating illness trajectories in critically ill patients.

Further, risk scores from predictive models use already available physiologic information and do not require additional testing. Information regarding the entropy illness trajectories during critical illness may be valuable for prognostication and clinical decision-making. Entropy values may be valuable for risk stratification on adverse outcomes of PICU admissions and may help determine which children might benefit from additional clinical surveillance.

Given the challenges associated with accurate prognostication and prognostic communication between clinicians and families during periods of critical illness, identifying additional ways to characterize illness trajectories is an important endeavor. The interpretation of an entropy score can be considered as follows: (1) patients with a high entropy score likely have multiple transitions between many illness states, and (2) patients with a low entropy score likely stay within their state of illness more often (which can be either remaining in a low severity state or remaining in a high severity state). Given this interpretation, an entropy score requires interpretation in conjunction with clinical and therapeutic correlation. A patient with high entropy in the PICU may represent a lower decision-making threshold for clinicians when determining whether or not to obtain a blood culture for concerns related to sepsis. One potential use case for a low entropy score could be for the pediatric patient who has been critically ill for many days in a high state of illness (with an associated high level of therapeutic intensity including mechanical ventilation, vasopressor support, etc.) with very few transitions out of the high state. The low entropy score, along with the clinical and therapeutic correlation, could be used to initiate consult with palliative care to anticipate the need for supportive care measures [27]. On the other end of the spectrum, a low entropy score in association with low severity of illness and low therapeutic intensity level could indicate that the patient could be ready for a successful discharge to the pediatric acute care ward or home. In this sense, both high entropy and low entropy scores are of interest when interpreted in conjunction with the clinical and therapeutic correlates.

There are limitations to this analysis. Data collection was limited to a single tertiary academic children’s hospital and was based on risk scores from a single predictive model. The results may not be generalizable to other settings and predictive models, particularly to models without validation of their calibration. Some challenges require further investigation before implementing this approach at the bedside. We used scores throughout entire PICU admissions to calculate entropy. The minimum duration of data necessary to calculate entropy requires further exploration. We chose to use Shannon entropy to quantify illness state transitions. This approach required creating discrete illness states. Sample entropy, which does not require discretizing illness states, may have value here. Additionally, as outlined in the examples above, the entropy score is not a stand-alone metric and should only be used in conjunction with measures of clinical illness severity.

## Conclusion

In this study, we developed a method to quantitatively characterize the dynamics of illness state transitions during admission to in intensive care unit among children with sepsis diagnoses. We found that the entropy of an illness trajectory is associated with negative clinical outcomes for children in the PICU. We are unaware of other studies that assess the association of entropy of an illness trajectory to clinical outcomes. This analysis supports extending current continuous predictive monitoring platforms with the development of individual trajectories based on patient’s entropy. Further study is needed to investigate the potential for applying an entropy score within the pediatric intensive care setting with annotation of events indicating responsiveness to therapy, supportive care interventions, and the relationship of entropy to long-term functional outcomes for PICU survivors. Understanding the dynamics associated with a child’s critical illness trajectory, including the transitions within the trajectory, provides a novel analytic lens with the potential for multiple clinical and research applications.

## Supporting information

S1 Fig A scatter plot to show the association between illness score and entropy. (TIF)
