# Supplementary figures and images for: Individual illness dynamics: An analysis of children with sepsis admitted to the pediatric intensive care unit
Source: PLOS Digit Health. 2022 Mar 17;1(3):e0000019. doi: 10.1371/journal.pdig.0000019 (PMC9931234; doi:10.1371/journal.pdig.0000019)

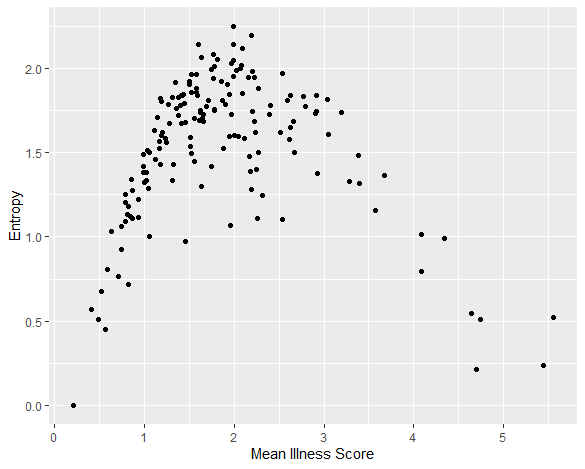

Supplement: S1 Fig — (TIF) [file pdig.0000019.s001.tif]
